# Effectiveness of a complex intervention in reducing the prevalence of smoking among adolescents: study design of a cluster-randomized controlled trial

**DOI:** 10.1186/1471-2458-14-373

**Published:** 2014-04-16

**Authors:** Alfonso Leiva, Andreu Estela, Maties Torrent, Amador Calafat, Miquel Bennasar, Aina Yáñez

**Affiliations:** 1 Primary Care Research Unit of Mallorca, Baleares Health services-IbSalut, Mallorca, Spain; 2 Dalt Sant Joan Health Centre, Baleares Health services-IbSalut, Menorca, Spain; 3 Àrea de Salut de Menorca, IB-SALUT, Menorca, Spain; 4 European Institute of Studies on Prevention (Irefrea), Mallorca, Spain; 5 Research Group on Evidence, Lifestyles and Health, Universitat Illes Balears, Palma, Spain; 6 Instituto de Investigación Sanitaria de Palma (IdISPa) & Research Group on Evidence, Lifestyles and Health, Universitat Illes Balears, Mallorca, Spain

**Keywords:** Adolescent, Intervention study, Randomized controlled trial, Risk-reducing behavior, Schools

## Abstract

**Background:**

The likelihood of an adolescent taking up smoking may be influenced by his or her society, school and family. Thus, changes in the immediate environment may alter a young person’s perception of smoking.

**Methods/Design:**

The proposed multi-center, cluster-randomized controlled trial will be stratified by the baseline prevalence of smoking in schools. Municipalities with fewer than 100,000 inhabitants will be randomly assigned to a control or intervention group. One secondary school will be randomly selected from each municipality. These schools will be randomized to two groups: the students of one will receive any existing educational course regarding smoking, while those of the other school will receive a four-year, class-based curriculum intervention (22 classroom lessons) aimed at reinforcing a smoke-free school policy and encouraging smoking cessation in parents, pupils, and teachers. The intervention will also include annual meetings with parents and efforts to empower adolescents to change the smoking-related attitudes and behaviors in their homes, classrooms and communities.

We will enroll children aged 12-13 years as they enter secondary school during two consecutive school years (to obtain sufficient enrolled subjects). We will follow them for five years, until two years after they leave secondary school. All external evaluators and analysts will be blinded to school allocation.

The aim of this study is to analyze the effectiveness of a complex intervention in reducing the prevalence of smoking in the third year of compulsory secondary education (ESO) and two years after secondary school, when the participants are 14-15 and 17-18 years old, respectively.

**Discussion:**

Most interventions aimed at preventing smoking among adolescents yield little to no positive long-term effects. This clinical trial will analyze the effectiveness of a complex intervention aimed at reducing the incidence and prevalence of smoking in this vulnerable age group.

**Trial registration:**

Current Controlled Trials: NCT01602796.

## Background

Tobacco use is a known risk factor for many chronic illnesses; it is the main cause of avoidable mortality in Europe, and it is responsible for 4.2 million deaths per year worldwide [1,2]. The prevalence of tobacco use among adolescents in the European Union is high, with approximately 15% of 14- and 15-year-olds smoking, although this percentage varies from 7% to 23% among countries [3]. Some countries have reported a change in the pattern of initial consumption in recent years, with more girls beginning to smoke, especially among younger age groups [4]. Within any given country, a high variability in smoking behavior is seen across schools [5-7].

The various factors associated with taking up smoking can be grouped into three categories, individual, social and cultural factors [8]. Individual factors are intrinsically linked to a given person and include the individual’s age and certain cognitive, emotional and biological aspects. The factors associated with the immediate social context (i.e., the environment closest to the adolescent) include the smoking-related attitudes and behaviors of family members and friends. The factors associated with the socio-cultural environment (i.e., the wider social context) include the smoking policy of the adolescent’s school.

Around 70% of current smokers began smoking before the age of 18 years, and those who do not begin to smoke by this age are highly unlikely to become smokers as adults [9]. Preventing young people from starting to smoke and helping young smokers break the habit are currently the most effective means to reduce the number of deaths attributable to smoking in the medium and long terms. To this end, various European countries have introduced programs designed to prevent tobacco use. Such programs should be based on scientific evidence and seek to reach children before they commence smoking.

A review of the effectiveness of distinct school- and/or community-based interventions to prevent smoking [10] indicates that multi-sector interventions (i.e. those involving the classroom, school, extracurricular activities, family, and community) yield more promising results than single-sector interventions. Furthermore, the evidence suggests that school-based preventive programs are not effective on their own; instead, interventions should be community-based, and should have impacts in the classroom, the school environment, and the community [11].

We propose to carry out a cluster-randomised controlled clinical trial to analyse the effectiveness of a multifactorial intervention addressed to adolescents and, in particular, their environment (educational centre, teachers and parents), as we believe that altering the adolescents’ immediate environment could have a considerable effect on reducing tobacco use among the young.

Cluster randomization is required because the interventions will be implemented at the school level.

## Methods

### Design and environment

This prospective multi-center cluster-randomized controlled trial will involve schools and communities stratified by school-level tobacco consumption at baseline. The clusters will consist of municipalities in the Balearic Islands. Control schools will continue to implement any smoking-prevention activities that are already in place when the study begins.

### Inclusion criteria

The Balearic island is organized into four insular councils (Majorca, Minorca, Ibiza and Formentera) and those in municipalities. There is 53 municipalities in Majorca and 8 in Minorca, between 300 to 350,000 inhabitants. All Balearic Island municipalities in Majorca and Minorca having fewer than 100,000 inhabitants and at least one public compulsory secondary-educational institution will be included. In municipalities containing more than one secondary school, the secondary school to be included will be determined through a randomization process. If the selected school declines to participate, another school from the same municipality will be invited to take part.

### Study population

The populations of the included municipalities vary from 11,000 and 30,000 inhabitants. The main economic activities of these communities are tourism and farming.

The study participants will consist of all 12-13 year-old students, who enrolled in the first grade of compulsory secondary education (ESO) over two consecutive academic periods (i.e., 2011-12 and 2012-13). The intervention will take place over the four years during which these adolescents are enrolled in ESO. Written informed consent will be obtained from all students and from at least one parent/guardian per student, according to the declaration of Helsinki.

### Pilot study

A pilot program is currently underway in a single public secondary education center to test the proposed intervention, the materials, and the data-collection networks. Following the four-year intervention and follow-up, data will be gathered and evaluated. We plan to implement the full study at the two-year mark of the pilot program.

### Recruitment

Headmasters at the selected schools will be contacted by a research team member, who will provide details about the study and invite the school to participate.

### Random assignment

Once informed consent and the commitment to participate have been obtained from the students/parents and the school, respectively, a baseline survey will be performed to determine the prevalence of smoking. Thereafter, the participating schools will be subjected to stratified randomization according to the baseline prevalence of tobacco use at each school by the “random” module, and the option separate strata of PEPI (version 4.0, Sagebrush Press).

### Measurements

Table 1 summarizes the study measurements, variables and schedule. The baseline questionnaire will gather data on a given student’s sociodemographic characteristics, environment and tobacco use, and the attitudes of his/her parents and teachers towards smoking. The smoking question was adapted from a previously validated questionnaire for adolescents [12]. Information on tobacco use will be collected through the following question: *Which of the following statements best describes you? (A) I have never tried to smoke; (B) I have tried cigarettes a few times, but I do not smoke now; (C) I currently smoke less than one cigarette per month; (D) I currently smoke at least one cigarette per month, but less than one cigarette per week; (E) I currently smoke at least one cigarette per week; (F) I smoke every day; (G) I used to smoke regularly in the past, but I do not smoke now.*

**Table 1:** Measures, questionnaires and timeline

**Instrument**	**Assessment area**	**Timing of assessment**
Pre-test questionnaire	Baseline prevalence of smoking	Before randomization
Baseline data form	Student smoking behaviors	At baseline
Teacher baseline data form	Teacher smoking behaviors and attitudes towards smoking and prevention	At baseline
Parent baseline data form	Parent smoking behaviors and attitudes towards smoking	At baseline
Smoking prevalence form	Prevalence of smoking among adolescents	After the fourth year of ESO and two years after graduation

### Intervention

ITACA (“multifactorial intervention to reduce the prevalence of smoking in the adolescent population: a cluster-randomized trial”) is a cognitive-behavioral intervention based on the social-influences model. It is designed to prevent adolescents from starting to smoke and to empower them to change the smoking-related attitudes and behaviors within their immediate environments, including their homes, classrooms and communities (e.g., neighborhoods, town squares and/or villages). This intervention will be integrated into the schools’ curricular activities. The main components of this intervention and the activities to be performed are summarized in Table 2.

**Table 2:** Intervention components

**Component of the intervention**	**First year (age 12-13 years)**	**Second year (age 13-14 years)**	**Third year (age 14-15 years)**	**Fourth year (age 15-16 years)**
**School-based interventions**	Seven lessons: two on information about the harmful effects of smoking; four on refusal skills (self-esteem, interpersonal relationship skills, problem-solving skills, ability to recognize risky situations, and strategies for accepting rules and limits); and one on identifying social influences that encourage people to start smoking, and smoking cessation	Six lessons: three about the harmful effects of smoking; one on refusal skills (interpersonal relationship skills and critical thinking); two on identifying social influences that encourage people to start smoking (tobacco company advertising campaigns, debunking false beliefs about tobacco), and smoking cessation	Five lessons: three on refusal skills (strategies for accepting rules and limits, problem-solving skills, group pressure, self-esteem and coping with emotion and stress); two on identifying social influences (debunking false beliefs about tobacco, and anti-advertising workshops designed to sensitize people to the power of advertising), and smoking cessation	Four lessons: one on the harmful effects of smoking; one on refusal skills (critical thinking and problem-solving skills); two on identifying social influences that encourage people to start smoking (tobacco company advertising campaigns, debunking false beliefs about tobacco), and smoking cessation
**Parental interventions**	Two work meetings focused on parental attitudes towards drug consumption and helping parents recognize risky situations, establish rules and limits, understand the family’s role in tobacco use and the smoke-free home initiative, and implement smoking cessation	Two work meetings focused on parental attitudes towards drug consumption and helping parents recognize risky situations, establish rules and limits, understand the family’s role in tobacco use and the smoke-free home initiative, and implement smoking cessation	Two work meetings focused on parental attitudes towards drug consumption and helping parents recognize risky situations, establish rules and limits, understand the family’s role in tobacco use and the smoke-free home initiative, and implement smoking cessation	Two work meetings focused on parental attitudes towards drug consumption and helping parents recognize risky situations, establish rules and limits, understand the family’s role in tobacco use and the smoke-free home initiative, and implement smoking cessation
	Collaborate with the student on homework regarding parental tobacco habits and attitudes, social norms and passive smoking	Collaborate with the student on homework regarding parental tobacco habits and attitudes, social norms and passive smoking	Collaborate with the student on homework regarding parental tobacco habits and attitudes, social norms and passive smoking	Collaborate with the student on homework regarding parental tobacco habits and attitudes, social norms and passive smoking
**Teacher interventions**	Teacher training on: competences related to smoking prevention in adolescents; managing conflict situations; attitudes towards preventive activities; information on the prevalence of smokers in the educational center; and smoking cessation	Smoking cessation.	Smoking cessation	Smoking cessation
**Smoke-free policy reinforcement**	The schools will be invited to adopt a smoke-free environment, implement the school’s own rules, and establish rule-compliance indicators			

#### ***Classroom sessions***

The four-year curricular component will consist of 22 lessons of approximately 50 minutes each, including eight lessons that will be presented as part of the environmental sciences curriculum, six as part of the social sciences curriculum, two as part of the physical education curriculum, one in the mathematics curriculum, and five that will be presented as student tutorials.

The components of the social-influences model that will integrated into this curriculum include teaching students to identify the social influences that encourage people to start smoking (e.g., tobacco company advertising campaigns, peer pressure), providing information about the harmful effects of smoking, and nurturing refusal skills. The curriculum will include anti-advertising workshops that are designed to sensitize participants to the power of advertising. The intervention will also seek to: debunk false beliefs about tobacco; encourage positive means for coping with emotion, stress and peer pressure; and help students develop interpersonal relationship skills, self-esteem, strategies for accepting rules and limits, critical thinking skills, problem-solving skills, and the ability to recognize risky situations.

The classroom sessions will be given by trained teachers in the intervention components, smoking prevention and Health. It will consist of seven lessons in the first year (ages 12-13), six lessons in the second year (ages 13-14), five lessons in the third year (ages 14-15) and four lessons in the fourth year (ages 15-16) of ESO. All lessons will be age-appropriate and relevant to the students’ curricula. All lessons will include material that students will work on together with their parents.

#### ***Families***

The parents of our participants will meet with the personnel of the study, at least once per year at the beginning of each academic to receive information about the intervention and learn how they can help keep their children from smoking. These meetings will focus on encouraging parents to: have an appropriate attitude towards drug use; recognize situations that constitute a risk for adolescents; establish rules and limits; be sensitive to the role that family plays in tobacco use; and participate in the smoke-free home initiative. At least once per year, a workshop on smoking cessation will be held for the participating families of each municipality.

One novel aspect of the intervention is that 20% of the curricular sessions will ask the student to involve their families in home-based tasks related to smoking prevention. Furthermore, a minimum of three leaflets will be provided each year regarding smoking in adolescents. The leaflets will contain answers to questions frequently asked by families, along with support material from the curricular sessions that parents and students can work on together. Parents will also receive information on make your home a smoke-free. Finally, parents will have access to a webpage that offers information on the school intervention, tips on how families can help prevent children from smoking, and advice on smoking cessation in adolescents.

#### ***Teachers and the school’***

Prior to the start of the intervention, the teachers of the enrolled schools will participate in training course. During this course, the needs of the teaching staff regarding smoking prevention and health themes will be identified. This course has been recognized by local government boards of education. Each school will designate a coordinator to carry out the intervention.

Classroom lessons will be subject to approval according to the school’s internal rules, and a communication system involving the coordinator, the teachers and the project researchers will be established. Annual meetings will be held with the school management team to reach agreements on the application of current legislation and the organizational structure of each smoke-free school. Teachers will participate in an initial on-line 20-hour training workshop. They will be instructed on skills related to smoking prevention in adolescents and management of conflict situations. This workshop will also address the teachers’ attitudes towards drug use and instruct them on preventive activities that will promote health.

The participating schools will be invited to adopt a smoke-free environment policy. The teachers at each school will hold a meeting to establish the school’s rules with respect to tobacco use by pupils and teachers. Each school will: implement its own rules; evaluate compliance; inform all teachers, students and parents of the changes being made to the rules; and establish a means to assess and record rule-compliance indicators, including incidents involving students and others smoking in the school.

The protocol for implementing the basic curriculum and the supplementary components of the intervention have been standardized in an effort to homogenize the intervention. The supplementary components include: the timing and contents of the lessons; agendas for the teachers’ meetings; the contents of the initial teacher-training workshop; the contents of the parental meetings; and the establishment of a professional advice network for parents and teachers who decide to quit smoking during the study.

### Strategies to ensure implementation of the intervention and avoid loss to follow-up

For the project to be successful, we will need to maintain our collaborations with the schools for at least four years. Therefore, all activities associated with the project must conform to the needs and interests of each school. Specific activities will be designed to promote continuity, including visits by the research team and periodic mailings to each school informing them of the progress of the study and recognizing their crucial role. The importance of the control group in the study will also be emphasized.

The final follow-up evaluation will occur six years after inclusion, when the participants will have left the participating school. At least three telephone contact numbers and the addresses of parents and tutors will be obtained early in the study. If a student is not easily located for the final evaluation, additional efforts will be made to contact them by telephone and letter. Efforts will be made to minimize the rate of loss to follow-up to reduce any possible bias resulting from loss to follow-up and to adequately assess the impact of the intervention.

#### ***Outcome assessments***

The measures, variables, and timeline are summarized in Table 1. The baseline prevalence of smoking in each school will be assessed by an external evaluator who will be blinded to the group (intervention or control) to which the school has been allocated. All outcome assessors and data analysts will also be blinded to school allocation. To evaluate the effectiveness of blinding, these individuals will be asked to choose the arm to which they believe each school was assigned (possible answers: intervention, control, or unknown). Individuals who respond “intervention” or “control group” will be asked to indicate what led to the formation of that belief. The primary outcome measure will be the prevalence of smoking at years three and six from enrollment. This will be assessed by self reports of tobacco consumption, where smokers are defined as those who consume more than one cigarette per week.

#### ***Statistical analysis***

##### 

**Sample size** A recent study on smoking in adolescents aged 14-15 years (third year of ESO) found that the prevalence of regular tobacco use in the Balearic Islands was 14% [13]. Using this as the prevalence for our control group and presuming that our intervention will reduce the prevalence of tobacco consumption in the intervention group by at least 40%, the use of 1,002 students per group will yield a statistical power > 80% for our between-group comparisons, assuming a bilateral α error of 5% and a 10% loss of participants to follow-up. The cluster size will be approximately 100 adolescents per municipality; we expect a 0.02 intra-class correlation coefficient [14], yielding a 2.98 cluster design effect. The design effect was calculated using the formula: Deff = 1 + (m – 1) * ICC; where “Deff” corresponds to the design effect, “m” is the cluster size and “ICC” is the intraclass correlation coefficient. The target sample size for each group was calculated to be 1,113 students, or 2,226 students in total. To achieve this sample size, students in the first year of ESO (aged 12-13 years) will be recruited over two consecutive years at 22 schools in the Balearic Islands.

##### 

**Analytic strategy** We will test for significant differences in baseline characteristics between the control and intervention groups. We will perform a descriptive and cluster analysis with continuous variables summarized using means and standard deviations for normal distributions, and by medians and 25^th^ and 75^th^ percentiles for non-normal distributions. All data analyses will involve intention-to-treat populations (i.e., all randomized patients, regardless of participation in any treatment session). This approach will reduce the bias that may occur when participants that fail to receive the assigned treatments are excluded from the analysis. All tests will be two-sided, and α-values of 0.05 will be considered statistically significant.

We will compare the smoking prevalence of adolescents in each group at 12 months against the usual null hypothesis of no difference between groups. We will use the Chi-squared test, taking into account the “variance inflation factor” of the adolescent cluster and intraclass correlation coefficient. We will also calculate 95% confidence intervals to assess the clinical significance of our intervention. In our multivariate analysis, we will adjust for potential confounders, if any, using a multilevel logistic regression model. We will estimate the relative and absolute risk reductions and the number needed to treat (i.e., the estimated number of adolescents who must be treated with our intervention rather than the existing school interventions for one additional adolescent to be prevented from smoking), which will be calculated as the reciprocal of the difference between the prevalence of adolescent smokers in the intervention and control groups. All estimates will include 95% confidence intervals.

### Ethical approval

Our study protocol has been approved by the Primary Care Research Committee of Majorca and the Balearic Island Clinical Research Ethical Committee (IB 1146/09 PI).

### Limitations

One of the main limitations of community studies is the possibility of contamination, such as would occur if teachers from neighboring schools met and compared notes regarding the intervention and/or control programs. To avoid this type of contamination, the randomization unit will be the municipality. Another possible limitation is that the intervention will be carried out among students who attend the same school. This could reduce the variability among study subjects; however, we will solve this by using a sufficiently large sample. There may also be a selection bias, with higher rates of non-participation by students attending the intervention schools compared to the control schools. However, in our pilot study implementing the intervention, the response rate to the first evaluation survey of tobacco use in 14 year olds was 85%.

Long-term follow-up of former students can be difficult because many youths move away from home and have life experiences that complicate follow-up. This may result in selection biases that could compromise the internal validity of the study. Consequently, efforts will be made to collect as many contact details as possible before students leave the school (e.g., the physical addresses of their parents, telephone numbers, e-mail addresses, and social network information). Multiple efforts will be made to maintain contact with former students and their families, thus minimizing the rate of loss to follow-up.

To guarantee the comparability of the control and intervention groups, the municipalities will be stratified according to the baseline prevalence of smoking in the school prior to the intervention.

## Discussion

In the European Union, the percentage of regular smokers quadruples (from 5% to 21%) between the ages of 12 and 16 years, with the smoking rate in the latter group being similar to that in adults [13]. The factors related to smoking include tobacco use among friends and older siblings, gender, availability of money, the intention to smoke in the future, low self-control in resisting the pressure to smoke, spending free time in bars, and low self-esteem [15-18]. Experts on smoking have therefore proposed interventions in which adolescents are given information about tobacco use and taught to identify and resist social influences. Most of these interventions, however, have not demonstrated the expected positive results in randomized clinical trials [19-25].

During adolescence, young people consider and explore a wide variety of options, contemplate an ideal world, and reflect on alternative political, religious, family and moral systems. The discrepancies between an adolescent’s ideal world and the real world are traditionally understood to form the basis for conflicts with the system, family, and/or moral imperatives. However, it is possible to take advantage of these capacities of adolescents and provide them with the necessary tools to effect changes in their environment.

Various studies have analyzed how adolescents are influenced by their immediate social contexts and socio-cultural environments. For example, teacher behavior, the educational center, and the attitudes of parents have all been associated with the initiation of tobacco use [7,26]. In addition, the prevalence of smoking among adolescents is lower when smoking is not permitted at home, especially if there is a complete ban; interestingly, this effect is independent of whether the parents are smokers [15]. Similarly, a cohort study [21] found that strong parental disapproval helped prevent smoking among adolescents, even after adjustment for parental smoking. These findings confirm that parental attitudes towards smoking are more important than the parents’ actual behavior. Similarly, the attitude of parents towards, and emphasis on compliance with, smoking rules at school are related to smoking by adolescents [26,27]. The ITACA program is designed to reach the family through its adolescent members.

The effect of the wider environment on smoking has also been analyzed. For example, several transverse studies have shown that the percentage of smokers varies widely among educational centers [5-7]. Furthermore, a longitudinal study involving 166 secondary schools in England [6] found that the school influenced the percentage of students that smoked, independent of the students’ characteristics. Thus, it appears that the social environment can explain a large part of the variability among schools.

The evidence indicates that school-based preventive programs are not effective alone, but rather should be community-based, and should involve the classroom, the school environment, and the community [11]. However, few studies have rigorously analyzed the effect of such broader interventions. Most of the existing evidence comes from direct classroom interventions, with only anecdotal evidence of the intervention and its effect on individuals and the school environment [10]. Therefore, we herein propose a multifactorial intervention aimed at adolescents through their environment (school, teachers and parents). We believe that modifying the students’ immediate environment could have a marked effect on smoking rates among young people.

## Competing interests

The authors declare that there is no competing interest.

## Authors’ contributions

AY, AE, MT, AC and AL collectively drafted the study protocol and sought funding and ethical approval. AE and AY designed the intervention and are responsible for managing the trial. All authors have critically commented on a draft manuscript and have approved the final manuscript.

## Pre-publication history

The pre-publication history for this paper can be accessed here:

http://www.biomedcentral.com/1471-2458/14/373/prepub
